# Life expectancy of different ethnic groups using death records linked to population census data for 4.62 million people in Scotland

**DOI:** 10.1136/jech-2016-207426

**Published:** 2016-07-29

**Authors:** Laurence Gruer, Geneviève Cézard, Esta Clark, Anne Douglas, Markus Steiner, Andrew Millard, Duncan Buchanan, Srinivasa Vittal Katikireddi, Aziz Sheikh, Raj Bhopal

**Affiliations:** 1 Usher Institute for Population Health Sciences and Informatics, University of Edinburgh, Edinburgh, UK; 2 Demographic Statistics, National Records of Scotland, Edinburgh, UK; 3 Department of Child Health, University of Aberdeen, Aberdeen, UK; 4 Public Health Science Directorate, NHS Health Scotland, Glasgow, UK; 5 Information Services, NHS National Services Scotland, Edinburgh, UK; 6 MRC/CSO Social and Public Health Sciences Unit, University of Glasgow, Glasgow, UK

**Keywords:** ETHNICITY, INEQUALITIES, MORTALITY, Life course epidemiology

## Abstract

**Background:**

Few countries record the data needed to estimate life expectancy by ethnic group. Such information is helpful in assessing the extent of health inequality.

**Method:**

Life tables were created using 3 years of deaths (May 2001–April 2004) linked to Scottish 2001 Census data for 4.62 million individuals with self-reported ethnicity. We created 8 ethnic groups based on the census definitions, each with at least 5000 individuals and 40 deaths. Life expectancy at birth was calculated using the revised Chiang method.

**Results:**

The life expectancy of White Scottish males at birth was 74.7 years (95% CI 74.6 to 74.8), similar to Mixed Background (73.0; 70.2 to 75.8) and White Irish (75.0; 74.0 to 75.9), but shorter than Indian (80.9; 78.4 to 83.4), Pakistani (79.3; 76.9 to 81.6), Chinese (79.0; 76.5 to 81.5), Other White British (78.9; 78.6 to 79.2) and Other White (77.2; 76.4 to 78.1). The life expectancy of White Scottish females was 79.4 years (79.3 to 79.5), similar to mixed background (79.3; 76.6 to 82.0), but shorter than Pakistani (84.6; 82.0 to 87.3), Chinese (83.4; 81.1 to 85.7), Indian (83.3; 80.7 to 85.9), Other White British (82.6; 82.3 to 82.9), other White (82.0; 81.3 to 82.8) and White Irish (81; 80.2 to 81.8).

**Conclusions:**

Males and females in most of the larger ethnic minority groups in Scotland have longer life expectancies than the majority White Scottish population.

## Introduction

Life expectancy is a useful measure of population health but rarely estimated for ethnic minorities due to lack of data. Among the few countries recording the ethnic group of the deceased, the USA,^1^ New Zealand^2^ and Australia^3^ showed substantial differences in life expectancy, usually favouring the ethnic majority. Country of birth is used as a proxy for ethnicity in some countries but misclassifies people who do not identify with the majority ethnic group in their country of birth. Examples include the increasing number of people born in the UK whose parents or grandparents were immigrants, or White British born in India who returned to the UK after Indian Independence.^4^ ^5^

Morris *et al*^6^ recently estimated the life expectancy of White, Asian and Black groups in England and Wales. Lacking data on the ethnicity of individual deaths, they used age-specific and sex-specific mortality rates for all output areas in England and Wales, deriving the proportion of the population in each of the three groups from the 2001 Census. They then created life tables modelling the effect on mortality rates of increasing proportions of the population being Asian or Black, adjusting for the effect of area deprivation. Rees *et al*^7^ drew on evidence of a relationship between self-reported health and subsequent mortality. They estimated age-specific and sex-specific mortality rates for 16 ethnic groups using the 2001 Census, where self-reported limiting long-term illness and ethnicity are recorded. The resultant mortality rates were then used to develop life tables. They also used a geographical weighting model. All three methods have clear limitations which the authors acknowledged.

For this article, we aimed to calculate life expectancies for larger ethnic minorities using a data set linking individual records in the Scottish 2001 Census, which records self-reported ethnicity, with subsequent death records.

## Methods

### Linkage, security and disclosure

The Scottish Health and Ethnicity Linkage Study (SHELS) successfully linked death records to the 2001 Census records of 4.62 million people, including their self-reported ethnicity. The methods have previously been described.^8^ Full ethical and other approvals for the linkage, data security and analyses were granted by the Scottish Multicentre Ethics Committee and the Privacy Advisory Committee of NHS National Services Scotland. The data sets were only made available to named researchers with appropriate clearance and training in a safe haven at National Records Scotland (NRS). All analyses and outputs followed the NRS Disclosure Control Guidance for SHELS and were cleared for release by its disclosure committee.

### Data and analysis

As recommended by the Office for National Statistics,^9^ to provide enough deaths to calculate life expectancy by ethnicity and sex, we based our life tables on deaths in the 3 years after the Scottish Census 2001, from 1 May 2001 to 30 April 2004. Our base denominator of 4.62 million people was corrected downwards if an individual was known to have died or left the UK. As the cohort only included individuals who completed the Census, deaths of individuals born or entering the country after 1 May 2001 were not included. The 2001 census reported on 14 different ethnic groups. For life expectancy estimates of reasonable precision, we needed at least 5000 individuals and 40 deaths in every male or female ethnic group. We thus report on the eight ethnic groups using the Census definitions and fulfilling these criteria.

We used the revised Chiang method (Chiang II) to calculate life expectancy at birth by ethnic group and sex, with 95% CIs.^10^ This is a robust method for calculating life expectancy estimates for geographical areas^11^ and specific populations.^12^ To create the life tables, populations and deaths were counted by ethnic group, sex and 5-year age bands (except for 0, 1–4 and >85 years). Following Eayres' recommendations, no substitutions were made for zero death counts in any cell.^13^

## Results

Table 1 shows the estimated life expectancies for males and females in each of the eight ethnic groups. In comparisons, only values where the 95% CIs do not overlap are reported as different, roughly equivalent to p<0.01.^14^ The life expectancy of White Scottish males at birth was 74.7 years, overlapping with Mixed Background (73.0) and White Irish (75.0) but shorter than Indian (80.9), Pakistani (79.3), Chinese (79.0), Other White British (78.9) and Other White (77.2).

**Table 1:** Life expectancy at birth, by sex and ethnic group in Scotland

	Males					Females				
	Total deaths	Total linked census 2001 population	Expectation of life at birth	Lower 95% CI	Upper 95% CI	Total deaths	Total linked census 2001 population	Expectation of life at birth	Lower 95% CI	Upper 95% CI
Scotland										
White Scottish	65 115	1 949 485	74.7	74.6	74.8	73 875	2 138 645	79.4	79.3	79.5
Other White British	4455	160 235	78.9	78.6	79.2	4840	174 750	82.6	82.3	82.9
White Irish	965	20 340	75.0	74.0	75.9	990	23 160	81.0	80.2	81.8
Other White	800	29 945	77.2	76.4	78.1	675	35 710	82.0	81.3	82.8
Any Mixed Background	65	5310	73.0	70.2	75.8	60	5800	79.3	76.6	82.0
Indian	55	6450	80.9	78.4	83.4	40	5890	83.3	80.7	85.9
Pakistani	100	12 930	79.3	76.9	81.6	50	12 700	84.6	82.0	87.3
Chinese	55	6530	79.0	76.5	81.5	45	6670	83.4	81.1	85.7

Total deaths (3 years of deaths occurring between May 2001 and April 2004) and total Census 2001 linked numbers are rounded to the nearest five for disclosure reasons.

The life expectancy of White Scottish females was 79.4, overlapping with Mixed Background (79.3) but shorter than Pakistani (84.6), Chinese (83.4), Indian (83.3), Other White British (82.6), Other White (82.0) and White Irish (81.0). Among males and females, the 95% CIs for Other White British overlapped with those of Indians, Pakistanis, Chinese and Other Whites indicating they were broadly similar.

## Discussion

To the best of our knowledge, this is the first time that life expectancies of different ethnic groups have been calculated in a European country using individual death records linked to self-reported ethnicity. We found that most of the larger ethnic minority groups had longer life expectancies at birth than the White Scottish majority, with the central estimates being longer by 2.5–7.9 years.

Our study's main strength is that it calculates the life expectancies of ethnic minorities using self-reported ethnicity rather than country of birth^5^ and the death records of individuals rather than indirect mortality estimates.^6^ ^7^ It has some weaknesses. We were notified about only some of the individuals who moved to other parts of the UK and none of those who moved elsewhere. Discrepancies could thus occur if the deaths of departing members of ethnic minorities were disproportionately missed, but there is little evidence for this.^15^ The age of some older people born outside the UK may not have been accurately recorded. The numbers were too small in several ethnic groups to calculate life expectancy and in the four non-White groups to allow stratification for examining the effects of socioeconomic status or country of birth.

Our estimates of life expectancy were slightly higher than those published by NRS for 2000–2002, with the differences being greater for males.^16^ The NRS figures were based on whole population estimates and all deaths registered in Scotland. Our cohort only included those who completed the Census with their ethnicity and were linked to NHS data via a unique identifier.^8^ Those who did not complete the Census were more likely to be male and people living in socially disadvantaged areas, care homes and prisons, all associated with lower life expectancy,^17^ with a similar profile for those who could not be linked.

Morris *et al*^6^ found that in England and Wales, overall Asian and White life expectancies were similar, but those of Blacks shorter. We had insufficient numbers in our study to assess the life expectancies of Africans or African-Caribbeans. Adjusting their models for socioeconomic circumstances increased the life expectancy estimates for Asians and Blacks, reflecting the higher average levels of disadvantage experienced by these groups in England and Wales. However, the situation in Scotland appears more complex. We know from other analyses that the socioeconomic profiles of the Asian ethnic minorities in Scotland vary according to the specific ethnic group and the socioeconomic measures used (see online supplementary table). In a paper on gastrointestinal disease in this cohort, ‘ethnic variations were mostly not much altered by socioeconomic or country of birth adjustment’.^18^ We think that if we had been able to adjust for socioeconomic status, it would have been unlikely to alter greatly the differences in life expectancy between the ethnic groups.

10.1136/jech-2016-207426.supp1 Supplementary table

Our results suggest that, among people living in Scotland in 2001, the three ethnic minorities of Asian origin, and Whites with other origins except Ireland, could have expected to live several years longer on average than the White Scottish majority. The relatively poor health of the White Scots may explain much of these differences. Between 2003 and 2010, life expectancy for Scotland as a whole was 4–5 years shorter than that in many other Western European countries, although somewhat longer than in Eastern Europe.^19^ White Scots living in England have higher mortality rates than any other immigrant group except the Irish.^20^ The Asian groups in Scotland had similar life expectancies to the Other White British. Thus, if Asians in Scotland had instead settled in countries such as Switzerland, France or England, the comparison with the majority population might not look so favourable.

The causes of Scotland's poor health record are a matter of continuing debate. Much evidence points to material disadvantage and air pollution in early years and culturally mediated behaviours including smoking, drinking alcohol and unhealthy eating, which may be less prevalent in some ethnic minority groups.^21^ The possibility that people who migrate to another country, particularly over long distances, may be intrinsically healthier on average than those they leave behind (the healthy migrant effect) may also play a part.^22^ However, as these estimates are based on the age-specific death rates of the cohort in 2001–2004, they are not projections of future mortality experience. The extent to which ethnic minorities retain their distinctiveness or converge in health-related culture and behaviour towards the majority will influence future trends.^23^

Since the available Scottish death records for the first decade of the 21st century did not include the ethnicity of the deceased, these analyses were only possible because death records were linked to the 2001 Census where ethnicity was recorded. A linked cohort based on the 2011 Census is planned, providing an opportunity for updating in due course. Since 2012, the ethnicity of the deceased is requested as part of the death registration process in Scotland, to the best of our knowledge, uniquely in Europe.^24^ Provided that refusal rates are not disproportionately high in certain ethnic groups, this should soon allow life expectancies to be compared on an ongoing basis.

In conclusion, linking death records to a population census recording self-reported ethnicity provides the basis for estimating the life expectancy of ethnic minorities. The longer life expectancies of the larger ethnic minorities in Scotland may reflect the poor average health of the White Scottish majority as much as the good health of minority groups. This highlights the scope for continuing health improvement across the whole population.

**What is already known on this subject**

- The life expectancy of different ethnic groups in a defined population has rarely been calculated due to a lack of suitable data. Where it has, substantial differences between groups have often been found, typically in favour of the ethnic majority.

**What this study adds**

- Linking the 2001 Census, which recorded ethnicity, to subsequent death records provided enough data to estimate life expectancy for eight ethnic groups in Scotland.
- Most of the larger ethnic minority groups had longer life expectancies than the White Scottish majority. This may be due to the relatively poor health of the White Scottish population as much as to the good health of ethnic minorities.
