# Supplementary material for: Life expectancy of different ethnic groups using death records linked to population census data for 4.62 million people in Scotland
Source: J Epidemiol Community Health. 2016 Jul 29;70(12):1251–4. doi: 10.1136/jech-2016-207426 (PMC5136685; doi:10.1136/jech-2016-207426)
Supplement: Supplementary table [file jech-2016-207426supp_table.pdf]

Supplementary table

Socio-demographic profile of the linked Census population by sex and ethnic group

Sex and ethnic group	N	Age at Census		Country of Birth	Scottish Index of Multiple Deprivation		Highest Qualification (individual)	NS-SEC[1] (individual)
		(mean, SD)		*UK born (%)*	*Most Deprived (%)*	*Least Deprived (%)*	*High (%)*	*Managerial (%)*
**MEN**								
White Scottish	1949485	38	(22)	99.1	20.1	19.8	24.4	45.6
Other White British	160235	42	(20)	95.3	8.0	29.6	48.4	64.3
White Irish	20340	45	(20)	98.4	22.2	21.7	36.8	57.3
Other White	29945	36	(21)	30.5	12.4	32.6	49.2	63.6
Any Mixed Background	5310	21	(18)	76.0	19.7	26.1	36.8	56.2
Indian	6450	31	(19)	48.4	9.7	38.5	50.9	55.1
Pakistani	12930	27	(19)	58.0	15.8	24.8	26.5	31.9
Chinese	6530	30	(18)	38.4	13.4	38.5	31.8	34.7

Sex and ethnic group	N	Age at Census		Country of Birth	Scottish Index of Multiple Deprivation		Highest Qualification (individual)	NS-SEC[1] (individual)
		(mean, SD)		*UK born (%)*	*Most Deprived (%)*	*Least Deprived (%)*	*High (%)*	*Managerial (%)*
**WOMEN**								
White Scottish	2138645	41	(24)	99.1	21.3	19.2	23.8	30.4
Other White British	174750	44	(21)	95.0	8.2	28.9	40.8	43.6
White Irish	23160	49	(21)	98.6	20.1	22.8	38.0	47.9
Other White	35710	37	(21)	26.4	10.8	33.4	51.3	47.0
Any Mixed Background	5800	24	(20)	74.5	18.7	27.3	37.8	40.0
Indian	5890	30	(19)	51.1	9.6	39.0	40.7	32.5
Pakistani	12700	26	(18)	60.5	15.4	24.6	22.8	12.8
Chinese	6670	31	(18)	33.9	12.1	39.1	33.3	23.9

Total Census 2001 linked numbers are rounded to the nearest 5 for disclosure reasons

1 UK National Statistics Socio-economic Classification
